# A Comparative Review of Mandibular Orthognathic Surgeries with a Focus on Intraoral Vertico-sagittal Ramus Osteotomy

**DOI:** 10.7759/cureus.1924

**Published:** 2017-12-08

**Authors:** Puhan He, Joe Iwanaga, Yuki Matsushita, Nimer Adeeb, Nitsa Topale, R. Shane Tubbs, Jingo Kusukawa

**Affiliations:** 1 Harvard School of Dental Medicine, Harvard University, Cambridge, USA; 2 Neurosurgery, Tulane Center for Clinical Neurosciences, Tulane University School of Medicine, New Orleans, USA; 3 Department of Orthodontics and Pediatric Dentistry, University of Michigan School of Dentistry, Ann Arbor, USA; 4 Neurosurgery, Louisiana State University, Shreveport, USA; 5 Basic Sciences, St. George's University, True Blue, GRD; 6 Neurosurgery and Structural & Cellular Biology, Tulane University School of Medicine, New Orleans, USA; 7 Anatomical Sciences, St. George's University, True Blue, GRD; 8 Neurosurgery and Ochsner Neuroscience Institute, Ochsner Health System, New Orleans, USA; 9 Dental and Oral Medical Center, Kurume University School of Medicine, Kurume, JPN

**Keywords:** temporomandibular joint (tmj), sagittal split osteotomy, intraoral vertical sagittal ramus osteotomy (ivsro), intraoral vertical ramus osteotomy, oral surgery, prognathism, condylar hyperplasia, orthognathic surgery, condylotomy, condylar fracture

## Abstract

Severe dentofacial deformities require both orthodontics and surgical management to repair. Modern mandibular orthognathic surgery commonly uses sagittal split ramus osteotomy (SSRO) and intraoral vertical ramus osteotomy (IVRO) methods to treat patients. However, complications like neurosensory disturbances and temporomandibular joint disorders are common following both procedures. In 1992, Choung introduced the intraoral vertico-sagittal ramus osteotomy (IVSRO) which led to a decrease in postoperative complications. The 'straight' IVSRO or Choung’s type II osteotomy has a 'condylotomy' effect that reduces iatrogenic temporomandibular joint symptoms and treats preoperative temporomandibular joint symptoms. This osteotomy type is especially applicable for prognathism with excessive flaring of the ramus and with temporomandibular joint dysfunction. The 'L-shaped' IVSRO or Choung’s type I osteotomy is indicated for patients with condylar hyperplasia and high condylar process fractures.

## Introduction and background

The first surgical correction of oral malocclusion was performed by Hullihen in 1849, paving the way for increased interest in orthognathic surgery. In the early 1900s, Blair led St. Louis to become the fertile ground for the development of this field. He coined the five classes of jaw deformities: mandibular prognathism, mandibular retrognathism, alveolar mandibular protrusion, maxillary protrusion, and open bite. His surgical technique is now known as the Blair-Kostecka osteotomy (1907). This procedure is used to correct prognathism by performing a horizontal osteotomy of the ramus above the lingula with a Gigli saw through a stab incision. Despite being a short procedure, complications range from partial or total relapse, open bite, and dislocations, to pseudarthrosis and irreversible nerve injuries. These problems mainly arise due to small contacting bony surfaces aggravated by the pull of the temporalis muscle [1]. Thereafter, new surgical techniques were pioneered to overcome the drawbacks in the Blair-Kostecka surgery. A notable example is Schossmann-Kazanjian’s oblique osteotomy, an extraoral approach that increases the contact area of the two bony segments [1].

Modern orthognathic surgery was pioneered in central Europe by Heinz Kole and Hugo Obwegeser [1]. Obwegeser developed the sagittal split ramus osteotomy (SSRO) in 1957, with the goal of avoiding skin incision and producing broad contacting bone surfaces [1]. Following that in 1968, Winstanley performed the first intraoral vertical ramus osteotomy (IVRO) to manage horizontal mandibular excess, distal segment advancement of less than 2 mm, and rotation [2].

Today, severe dentofacial deformities require both orthodontics and surgical management to fix. The most commonly used orthognathic surgery of the mandible includes SSRO and IVRO [3]. If setback or advancement requires screws or metal plates, SSRO is preferred. This method showed stable results with rigid fixation (RF) [4]. Neurosensory disturbances (NSD) of the inferior alveolar nerve (IAN) are common following SSRO, especially when the posterior margin of the ramus curves inwards and the ramus is thin [5]. Comparatively, IVRO is primarily used for mandibular set back and not for advancement [7-8]. IVRO is preferred for rotational mandibular asymmetry because it causes less rotational displacement than SSRO. Rotational displacement could cause condylar displacement and temporomandibular disorder (TMD) [6]. Similar to SSRO, NSD is common following IVRO. Both SSRO and IVRO have the potential for postoperative condylar displacement [9].

**Figure 1:**
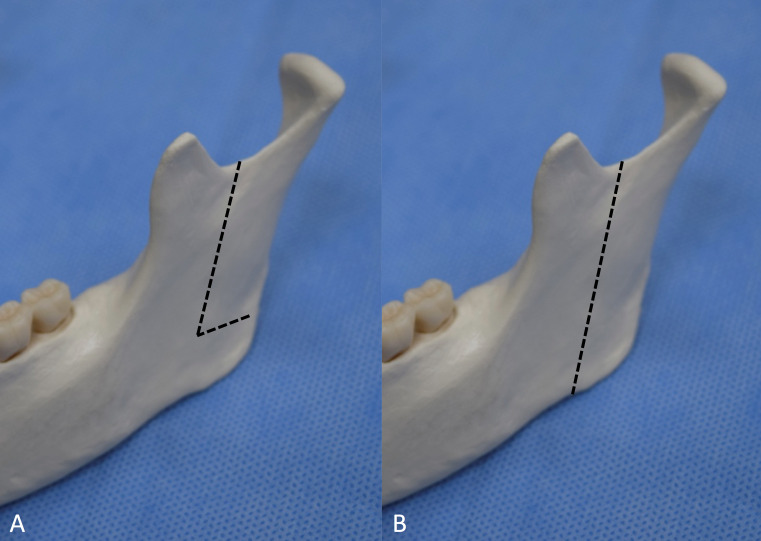
Lateral view of the osteotomy line A: ‘L-shaped’ intraoral vertico-sagittal ramus osteotomy. B: ‘Straight’ intraoral vertico-sagittal ramus osteotomy.

In 1992, Choung introduced the intraoral vertico-sagittal ramus osteotomy (IVSRO), combining the advantages of both SSRO and IVRO [3]. In his paper, Choung developed two different techniques; the ‘L-shaped’ IVSRO (Choung’s I osteotomy) and ‘straight’ IVSRO (Choung’s II osteotomy) (Figure 1). This review aims to discuss the conception and methods employed for this technique.

## Review

IVSRO techniques

Surgical Anatomy

**Figure 2:**
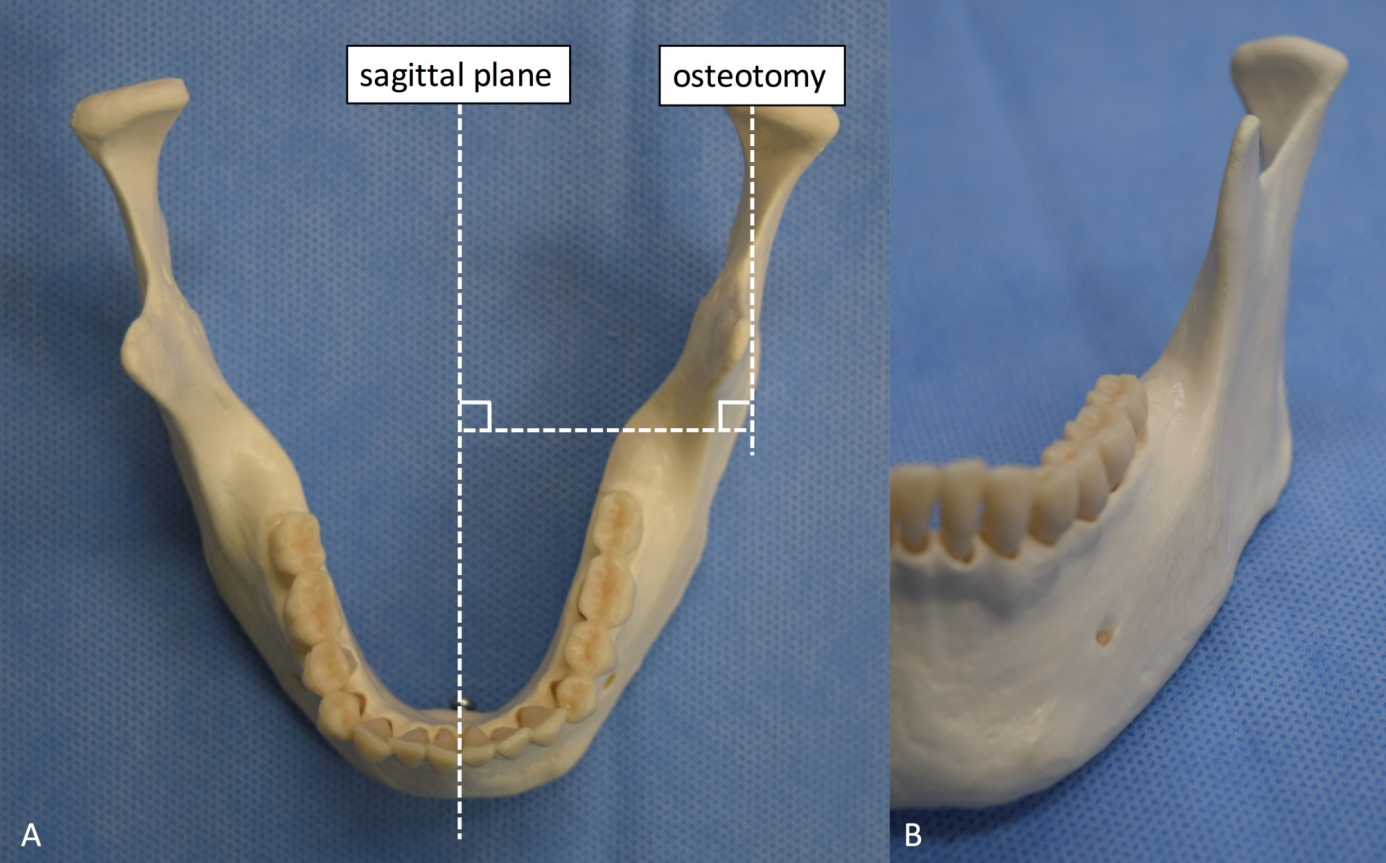
Original sagittal plane and vertico-sagittal ramus osteotomy plane A: Superior view. B: Anterior view.

**Figure 3:**
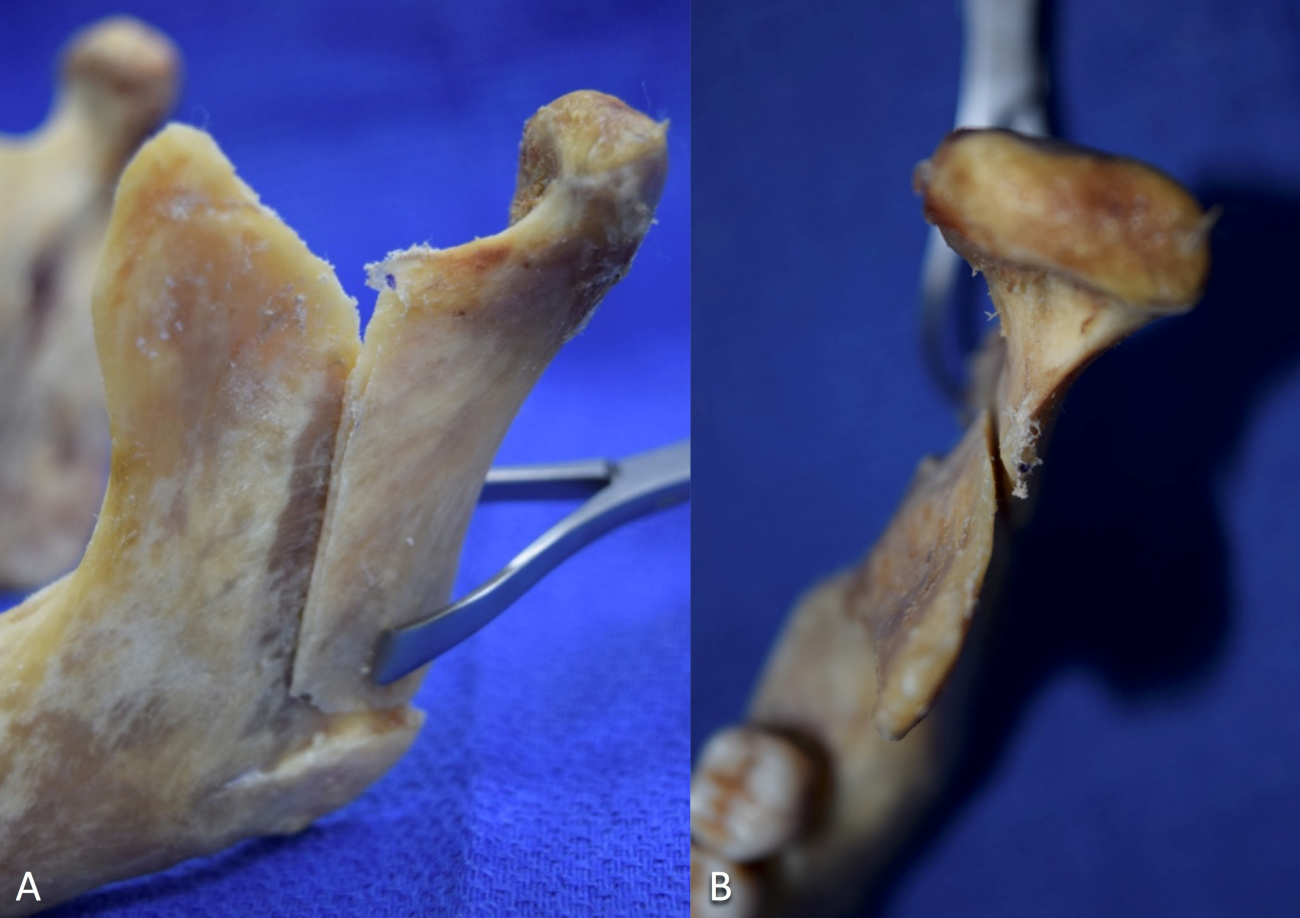
Example of the ‘L-shaped’ intraoral vertico-sagittal ramus osteotomy on a dry mandible A: Lateral view. B: Superior view.

IVSRO was developed to avoid displacement of the condyle by making an osteotomy plane that is parallel to the original sagittal plane (Figures 2-3). In order to make a parallel plane, the osteotomy plane is made in the posterior part of the ramus, from just anterior to the 'antilingular' prominence to the medial posterior border of the ramus. During this procedure, the mandibular canal is protected by a thicker trabecular bone compared to that of the anterior margin area of the ramus made in the SSRO technique. Therefore, IVSRO is less likely to cause IAN damage compared to SSRO [9].

**Figure 4:**
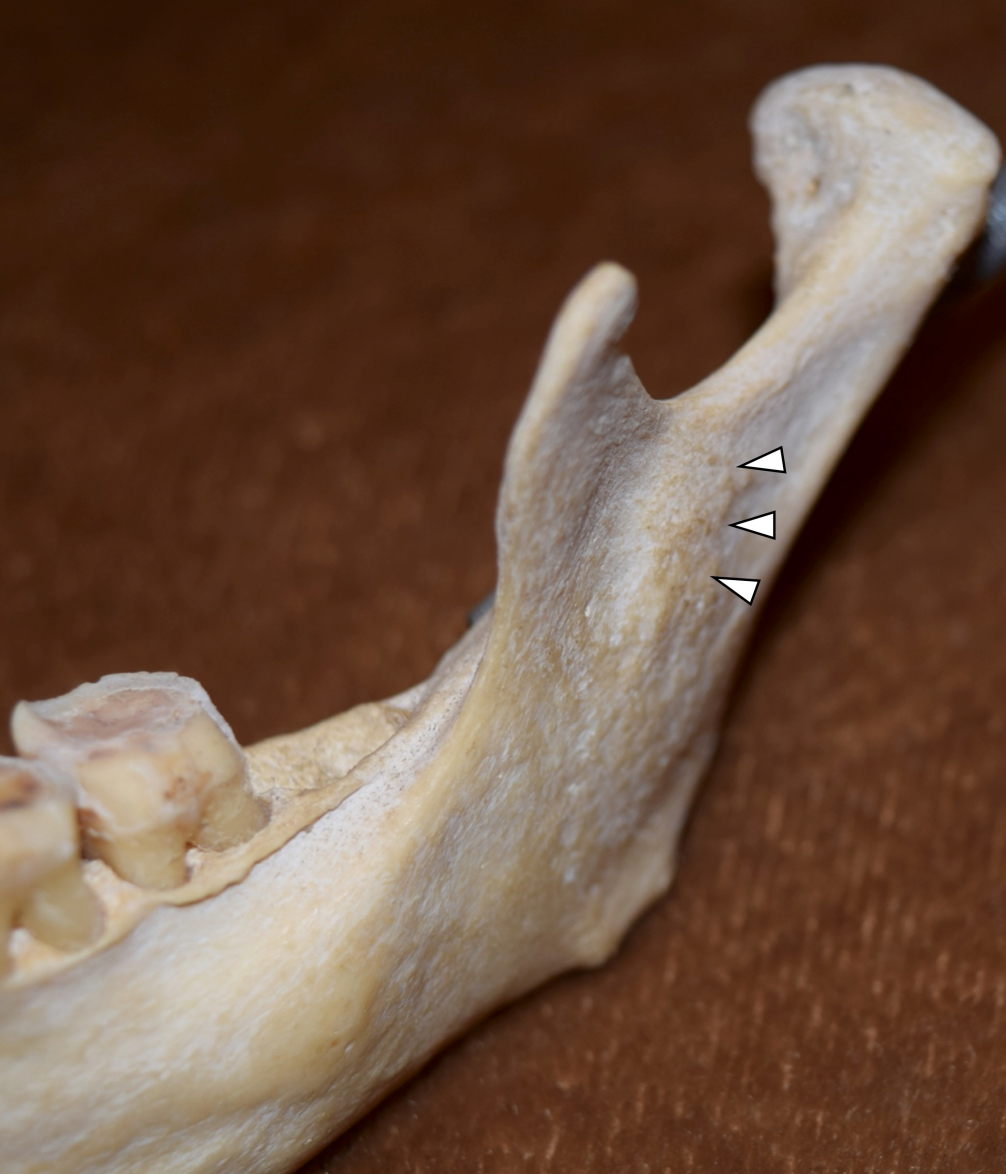
Antilingular prominence (arrowhead)

Having the ability to locate the IAN accurately intraoperatively is of utmost importance. The 'antilingular' prominence is thought to be a bony elevation on the lateral surface of the mandible marking the entry of the IAN on the medial side (Figure 4) [10]. Since Caldwell and Letterman first proposed this relationship in 1954, surgeons have been using this prominence to guide mandibular ramus osteotomies [11]. By placing osteotomies posterior or superior to this landmark, IAN injuries are presumably avoided. However, despite the prevalent use of this landmark as a proxy for IAN entry on the medial side, it is not without flaws. Three separate investigations revealed that the 'antilingular' prominence is only definitely present in 44% of the dried mandibles studied [12]. More importantly, only 18% of the prominences were within 3 mm of the mandibular foramen on the medial side [12]. Finally, it was shown that this lateral bony mandibular protuberance serves as an insertion point for muscles and tendons in the mandible [10]. Difficulties in identification as well as variable distances make it an unreliable landmark to safely guide surgeries.

Surgical Technique

**Figure 5:**
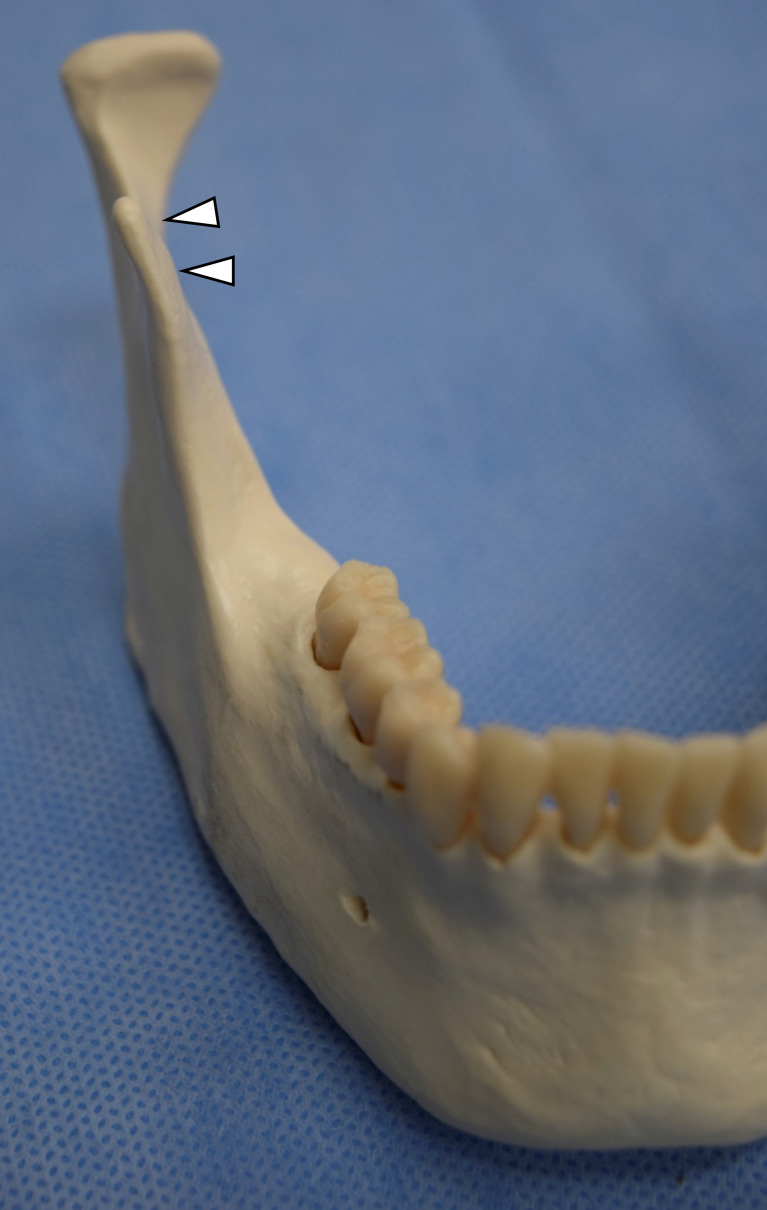
Laterally divergent coronoid process (arrowhead) Note the sigmoid notch; it cannot be seen in the view from the sagittal plane.

With the 'straight' IVSRO, the osteotomy plane, which theoretically lies parallel to the original sagittal plane, is created in the lateral aspect of the ramus. The proximal segment can be split straight without a horizontal osteotomy from the midsigmoid notch to the antegonial notch. The sagittally split proximal segment should contain the bony surface attached to the medial pterygoid muscle and the surrounding periosteum. The inner aspect of the lateral cortex of the proximal segment is overlapped on the decorticated distal segment. In cases where splitting of the lateral cortex cannot be performed easily, horizontal cutting of the medial cortex, as in the SSRO, can facilitate the splitting. Moreover, if the coronoid process is too divergent laterally to allow an approach to the sigmoid notch, the coronoid process may be sectioned (Figure 5). Once the coronoid process is sectioned, sagittal osteotomy can start from the sigmoid notch area, reflecting the coronoid process and the soft tissues [9].

In the 'L-Shaped' IVSRO, Bauer retractors are used to expose the lateral surface of the mandible from the sigmoid notch to the antegonial notch. A flat fissure bur is used to decorticate from the mid-sigmoid notch area inferiorly to the 'antilingular' prominence, then to the antegonial notch. An L-shaped osteotomy with partial thickness cutting is achieved by making a vertical osteotomy cut from sigmoid notch to the level of the mandibular angle, then a horizontal osteotomy cut in the posterior border of the ramus parallel to the occlusal plane is performed [9]. Iwanaga, et al. developed the modified ‘L-shaped’ osteotomy to reduce the chances of postoperative intraoperative hemorrhage and nerve dysfunction [13].

Stabilization Techniques

In the original description of IVSRO, the mandible was positioned with an interocclusal splint followed by intermaxillary fixation for six weeks. The 'straight' IVSRO does not require fixation because the proximal segment can be stabilized by the pedicle of the medial pterygoid muscle and the periosteum. Fixation methods vary for cases with TMD and those without for the 'L-shaped' IVSRO. For patients with TMD, the proximal segment was not fixed thus allowing passive movements. In patients without TMD, wires, screws or resorbable sutures were used to fix the proximal segments [9].

IVSRO indications

Choung reported that the IVSRO technique should be used for the treatment of condylar hyperplasia to reduce high condylar fractures and in patients with excessively flaring mandibles and temporomandibular joint (TMJ) symptoms [9, 14]. Other indications and applications are listed in Table 1.

**Table 1:** Comparison of different applicability of sagittal split ramus osteotomy, intraoral vertical ramus osteotomy, and intraoral vertico-sagittal ramus osteotomy RF: Rigid fixation

Application	Sagittal split ramus osteotomy	Intraoral vertical ramus osteotomy	Intraoral vertico-sagittal ramus osteotomy
Suitable for mandibular advancement	YES	NO (difficult to achieve adequate bone contact)	YES
Temporal mandibular joint disorder	NO	YES (condylotomy effect)	YES (condylotomy effect)
Internal RF necessary	YES	UNABLE	NO
Large contact area necessary	YES	NO	YES
Condylar hyperplasia	NO	NO	YES

Condylar Hyperplasia

Facial asymmetry and malocclusion are often undesired presentations of condylar hyperplasia [15]. Condylar hyperplasia is a condition with unknown etiology that is most commonly present in adolescents, and is characterized by the excessive growth of one mandibular condyle [15]. Consequent facial asymmetry can be vertical, horizontal, or a combination of both [16]. Surgical treatment is opted for in cases where the chin deviates 10 mm or more from the midline, or in cases where condylar growth is active [17]. The treatment includes high condylectomy with or without orthognatic surgery [15]. However, often times, orthognatic surgery is unable to correct the vertical gap between the condyle and glenoid fossa which results from the condylectomy, and thus recontouring and repositioning are required. Having multiple surgeries puts the patients at higher risks of further complications. Thus, Choung in 1998 proposed the use of IVSRO to treat cases with condylar hyperplasia allowing all procedures to be performed in one surgery. An 'L-shaped' IVSRO is performed to extract the affected ramus segment including the hyperplastic condyle and allows for the extra-corporal recontouring of the condyle with drills. At the same time, correction of malocclusion and asymmetries with additional osteotomies can be performed. Repositioning the condyle superiorly may be required to compensate for the gap. The recontoured condyle is stabilized into the articular fossa with osteosynthesis plates [14-15]. A five-year follow-up with this method showed no resorption or necrosis and also treated preoperative TMD, the presenting facial asymmetry, and masticatory difficulties [14].

Reduce High Condylar Fractures

In high condylar fractures, an 'L-shaped' osteotomy is performed to remove the fractured fragment. This segment can be externally reduced, fixed with wires or screws, and if necessary recontoured in the case of deformity and resorption. This allows for better vision and control, after which the condyle is reinstalled in a similar method to condylar hyperplasia treatment [14].

Using IVSRO to treat condylar hyperplasia and high condylar fractures allows for an intraoral approach. This minimizes IAN damage and allows for precise reduction and recontouring of the condyles extracorporeally. Although a similar replantation approach can be performed after SSRO and IVRO, the former produces a condyle segment that is overly large, while the latter has a contact surface that is too small [14]. 

IVSRO, SSRO and IVRO advantages and complications

Advantages

IVSRO was developed to have an osteotomy plane parallel to the original sagittal plane to prevent condylar displacement, a complication in SSRO and IVRO [9]. Furthermore, the osteotomy aims to prevent exposure of the IAN and medial ramus to limit IAN injury. Additionally, this technique would prove to be superior to IVRO and SSRO with its condylotomy effect, effectively reducing TMJ symptoms as presented in Table 2 [9,18].

**Table 2:** Comparison between the three different mandibular orthognathic surgeries

Complications	Sagittal split ramus osteotomy	Intraoral vertical ramus osteotomy	Intraoral vertical-sagittal ramus osteotomy
Neurosensitivity disturbances	HIGHEST	MEDIUM	LOWEST
Unfavorable splitting	LOWER	NO DATA	HIGHER
Horizontal relapse	VARIABLE	NO DATA	HIGH for > 5mm
Condylar displacement	MOST	MEDIUM	LEAST

NSD

NSDs are the most common complication following orthognathic surgery [19]. At least one year is typically required to have complete resolution of an NSD. It is particularly important for patients with prognathism. Fujimura, et al. observed differences with a p-value < 0.1 between dry mandible controls without prognathism and patients with prognathism. In the latter group, the mandibular foramen was located more posteriorly to the center of the width of the ramus and the tip of the lingula was more superior. Notably, there was also variable thickness of the ramus at the lateral margin of the mandibular foramen ranging from 0.5-7 mm (averaging 3.1 mm) in dry mandibles and 2-7 mm (averaging 4.4 mm) in patients with prognathism. There was also a difference between the ramus angles of the dry mandibles (11-22 degrees, average 16.8 degrees) and patients with prognathism (10-28 degrees, average 19.9 degrees). Because of the variation between individuals and in patients with prognathism, Fujimura, et al. recommends preoperative axial computed tomography (CT) or 3D-CT to accurately determine the distance between the mandibular foramen to the lateral cortical plate and the ramus angle especially for SSRO and IVSRO [20].

A comparative analysis between different orthognathic surgeries (Le Fort I and II, IVSRO, and SSRO), showed that the type of surgery was the most significant factor in nerve recovery variation [19]. Compared with the maxillary counterparts, mandibular surgeries cause longer recovery time and more severe neurosensory alterations. Furthermore, larger diameter nerve fibers are more prone to NSD and often have slower recovery patterns. In mandibular surgeries, trauma to the nerve often occurs during incisions and the subperiosteal retraction of soft tissues [19,21]. The retraction on the medial side of the mandibular ramus during SSRO results in demyelinating lesion secondary to compression [22]. The percentage of NSD after SSRO ranges from 20%-85% with complete resection in 2%-3.5% of the cases [23-28]. Comparatively, IVRO is less likely to cause nerve postsurgical neurosensitivity with a reported incidence range of 8%-36% [29-32].

Compared with SSRO and IVRO, IVSRO has a lower incidence of IAN damage, most likely because it avoids the mandibular canal. The split is made parallel to the original sagittal plane between the lateral cortical bone anterior to the 'antilingular' prominence and the mandibular canal [5]. This osteotomy is performed without exposing the IAN and can be done without exposing the medial side of the ramus. Moreover, because the coronoid process is divided, the osteotomy is easier to perform [9].

One case report from 1995 and 2005 showed that out of 237 patients who underwent IVSRO for mandibular setback, 7.2% had unilateral nerve exposure while 3.8% reported unilateral NSD of the IAN. Three out of nine of the patients who reported unilateral NSD also had nerve exposure and four out of nine patients reported intraoperative bleeding [33]. In another case series, 40 patients underwent bilateral IVSROs and reported no nerve injury [34]. The incidences of long-term NSD of the lower lip and chin after IVSRO was reported to be 0%-6% [5].

Furthermore, IVSRO is preferred for patients that require rigid fixation, as is the case when the posterior margin of the ramus curves inward or the ramus is thin. Although IVSRO does not require rigid fixation, in these cases rigid fixation can further reduce NSD. Damage to the IAN is limited because the osteotomy is in front of the foramen between the mandibular canal and immediately medial to the lateral cortical bone [5, 9]. NSD is also observed with IVRO [28, 32]. However, rigid fixation involves more technical difficulty and rotation of the condyle to the lateral side [9, 32]. IVSRO on the other hand has flatter, larger contact areas with more optimal medulla-to-cortex healing than the cortex-to-cortex healing in IVRO [5,9]. In SSRO, the excess overlap of the anterior edge of the proximal segment must be removed to fix the two segments together and to prevent distal rotation of the proximal segment [5]. In IVSRO, there is no excess overlap of the proximal segment.

One major critique in the measuring of postoperative NSD is the subjective nature of the tests. To improve upon previous tests, Park, et al. measured the current perception threshold of 186 patients to compare different orthognathic surgeries (Le Fort I, Le Fort II, SSRO, and IVSRO). This method uses a neurometer to objectively quantify the depolarization times of peripheral nerve fibers upon electrical stimulation [19]. The result showed that SSRO caused more NSD immediately after surgery, and at each follow-up test (three months, six months, and 12 months). After 12 months, IVSRO postsurgical values returned to presurgical ones, however SSRO did not. Moreover, Aβ fibers showed the largest current perception threshold value for all mandibular ramus osteotomy types. Interestingly, the combined genioplasty-SSRO procedure had significantly increased current perception threshold values for all nerve fibers (Aβ, Aδ and C fibers) while the combined genioplasty-IVSRO procedure only affected Aβ. However, the effect was not statistically as great as the effect of the main osteotomy type, thus the surgeon should still use combined methods when it yields better results [19].

Condylar Displacement

Orthognathic surgery often results in condylar displacement. The position of the condyle within the glenoid fossa is affected after mandibular setback surgery [35-36]. Notably, there is increased condylar displacement with the application of rigid internal fixation than when compared to wire fixation [37-38].

Postoperative condylar displacement has the disadvantage of both SSRO and IVRO. If there is excessive flare of the ramus, the osteotomy plane diverges more from the original sagittal plane in SSRO, which results in a medial displacement of the condyle. In IVRO, the result is a lateral displacement [9]. The prevalence of this complication ranges from 13% to 72% and leads to iatrogenic TMD [39-40]. In IVSRO, there is minimal condylar displacement. IVSRO minimizes condylar displacement because the osteotomy plane is parallel to the original sagittal plane. Although the condyle still experiences displacement in IVSRO because of difficulty in controlling the proximal segment, compared to SSRO and IVRO, there is no mediolateral displacement. Mediolateral displacement has a more severe consequence as compared to rotational changes in the condyle [9].

In a case series of 60 patients who underwent bilateral IVSRO with wire fixation, the average setback was 5.89 mm (range 3-8 mm) [41]. The osteotomy showed an increase in intergonial width (2.25 mm) and inter-ramus width (4.45 mm) as well as an increase in outward angulation of the condylar fragment (right ramus angles increased by 1.70 degrees and the left by 1.43). All these values positively correlated to the amount of mandibular setback. Bayat, et al. noted that to minimize outward angulation of the ramus, the medial pterygoid muscle should be preserved on the proximal segment and stripping of the masseteric tuberosity should be minimized [41].

Postoperative Iatrogenic and Preoperative TMD

Studies show that 14% to 97% of patients have concurrent TMD along with their orthognathic surgical needs [42-45]. One of the benefits of IVRO and IVSRO is the ‘condylotomy effect’ for treating TMJ symptoms that occur in conjunction with mandibular prognathism [9]. This effect defines the change in relationship of the condyle and articular disc that occurs postsurgically, improving TMD [34].

IVRO also has been used to treat patients with TMD symptoms [7-8]. However, because of the small contact area between the proximal and distal surface, IVRO is difficult to implement in patients that have a setback of more than 10 mm, or who require advancement, horizontal and/or vertical rotation of distal segment [3,18]. Instead of resorting to SSRO, which does not treat TMJ symptoms and causes iatrogenic TMD, IVSRO has been shown to reduce preoperative TMD and to reduce incidences of iatrogenic TMD [9]. IVSRO has a condylotomy effect. Specifically, there is anterior-inferior repositioning of the condyle, increasing the articular space and thus alleviating the burden on the glenoid fossa as well as bettering the articular disc-condyle relationship [18].

In a study by Fujimura, et al. on 34 sides with TMD symptoms, an IVSRO one-year follow up showed 94.1% of patients had improvement of TMJ sounds, mouth opening decreased from 49.7 mm to 44.6 mm and seven out of nine sides with preoperative joint or masticatory muscle pain had improvements. The study concluded that IVSRO is indicated for patients who had a brief period of abnormality of disc position and the procedure can treat TMJ sounds and pain in advanced and long-term TMD [18]. In another case series, 100% (14/14) of TMJ symptoms were improved with no iatrogenic TMJ pain [9].

Movements and Relapse

Sagittal and vertical repositioning of the condyle in the glenoid fossa after different orthognathic surgery varies. Relapse is associated with condylar distraction, position of the proximal segment, and the use of skeletal fixation [46-47]. Furthermore, there is a positive correlation with the amount of mandibular setback and the postoperative displacement of the condyle [33, 41]. Horizontal relapse at the supramentale point on cephalometric analysis in bilateral SSRO with RF varies from 0.51 mm to 2.85 mm [48-50].

In the case study of 237 patients who underwent IVSRO, the mean horizontal setback was 7.99 mm (range 2.5-13.2 mm) at the supramentale point at the time of release of mandibulomaxillary fixation. At one year follow-up, the horizontal relapse was 2.16 mm with a minimum of 0.8 mm and maximum of 4.5 mm. Hashemi, et al. concluded that patient age and sex had no correlation with the amount of relapse, whereas the amount of surgical setback had a positive correlation [33]. Another study of 22 patients who underwent IVSRO had a mean distal segment 8 mm setback, 4 mm in advance and 5 mm counterclockwise. Because of the correlation of the amount of setback to the amount of relapse, IVSRO is unsuitable for an operation that requires more than 5mm of mandibular advancement [3].

Bleeding

The incidence of bleeding was 9.3% in the study by Hashemi, et al. with 237 patients who underwent IVSRO [33]. According to Fujimura, et al. (2006), the lateral view of the maxillary artery was observed in cadaveric mandibles. The maxillary artery traverses superiorly and across the surface of the lower head of the lateral pterygoid muscle, nearing the ramus. Interestingly, there is variability in the course of the maxillary artery between different ethnicities. In 90% of Japanese patients, the maxillary artery traverses laterally and superficially to the lateral pterygoid muscle [20]. These values were stated to be 9%-55% in whites and 69% in blacks [20]. The distance of the maxillary artery from the midsigmoid notch was 3 mm in cadaveric mandibles. However, a branch of the maxillary artery, the masseteric artery, courses directly above the sigmoid notch entering the masseter muscle. Surgeons, therefore, must be careful when exposing the medial aspect of the sigmoid notch with the use of a retractor to prevent damage to the maxillary artery [20].

Procedural Time and Mandibulomaxillary Fixation Period

A bilateral IVSRO operation can be finished in 45 minutes [33]. The broad contact surface between the distal and proximal segments of the IVSRO compared to IVRO allows the mandibulomaxillary fixation period to be shorter. The mandibulomaxillary fixation period for an IVSRO procedure was 16 days according to Fujimura, et al. as compared to 21 days for an IVRO procedure in the same institution. In the least severe of patient cases, mandibulomaxillary fixation can be minimized to seven days [18, 34].

Unfavorable Split

Unfavorable splitting is when there is a separation of only one part of the lateral cortex. There was a higher incidence of an unfavorable split in IVSRO compared to SSRO. The SSRO incidence of unfavorable split ranged from 1.9% to 2.2%. However, in 237 patients study who underwent IVSRO, the incidence was 11% [33].

## Conclusions

SSRO and IVRO are commonly used to correct mandibular prognathism. Both procedures could result in condylar displacement. SSRO has a large contact area; however, this procedure has high incidences of NSD and does not relieve TMJ symptoms. Comparably, IVRO relieves TMJ symptoms and has a lower rate of NSD, but also has a small contact area resulting in longer treatment times and less stability. Choung’s Type I osteotomy is indicated for patients with condylar hyperplasia and high condylar process fractions. Choung’s Type II osteotomy has the advantages of both SSRO and IVRO; it decreases the incidence of NSD, has a large contact area, and relieves TMJ symptoms. It also offers the advantage of minimizing condylar displacements. These advantages are achieved because: 1) the osteotomy plane is parallel to the original sagittal plane, minimizing condylar displacement, 2) the osteotomy does not expose the IAN or the medial side of the ramus, and 3) the condylotomy effect reduces iatrogenic TMJ symptoms and treats preoperative TMJ symptoms. Choung’s Type II is thus highly suggested when treating dentofacial deformities associated with TMD.

Future studies should focus on some of the observed complications of IVSRO such as bleeding, ramus flaring, and transverse displacement of the proximal segments. As IVSRO has been proven to be effective in cases where IVRO cannot be used, and because SSRO should be avoided due to TMJ and risk of an IAN injury, IVSRO should become the standard for this subset of patients to achieve satisfying esthetics and functional results.
